# Multimodal Photoplethysmography-Based Approaches for Improved Detection of Hypertension

**DOI:** 10.3390/jcm9041203

**Published:** 2020-04-22

**Authors:** Kaylie Welykholowa, Manish Hosanee, Gabriel Chan, Rachel Cooper, Panayiotis A. Kyriacou, Dingchang Zheng, John Allen, Derek Abbott, Carlo Menon, Nigel H. Lovell, Newton Howard, Wee-Shian Chan, Kenneth Lim, Richard Fletcher, Rabab Ward, Mohamed Elgendi

**Affiliations:** 1 Faculty of Medicine, University of British Columbia, Vancouver, BC V6T 1Z3, Canada; kaylie.welykholowa@alumni.ubc.ca (K.W.); manish.hosanee96@gmail.com (M.H.); gabriel.chan@alumni.ubc.ca (G.C.); rachelcooper@alumni.ubc.ca (R.C.); weeShian.Chan@cw.bc.ca (W.-S.C.); klim@cw.bc.ca (K.L.); 2 Research Centre for Biomedical Engineering, City, University of London, London EC1V 0HB, UK; p.kyriacou@city.ac.uk; 3 Research Center of Intelligent Healthcare, Faculty of Health and Life Science, Coventry University, Coventry CV1 5FB, UK; ad4291@coventry.ac.uk; 4 Population Health Sciences Institute, Faculty of Medical Sciences, Newcastle University, Newcastle upon Tyne NE2 4AX, UK; john.allen@newcastle.ac.uk; 5 School of Electrical and Electronic Engineering, The University of Adelaide, Adelaide SA 5005, Australia; derek.abbott@adelaide.edu.au; 6 Centre for Biomedical Engineering, The University of Adelaide, Adelaide SA 5005, Australia; 7 School of Mechatronic Systems Engineering, Simon Fraser University, Burnaby, BC V5A 1S6, Canada; cmenon@sfu.ca; 8 Graduate School of Biomedical Engineering, UNSW Sydney, Sydney NSW 2052, Australia; n.lovell@unsw.edu.au; 9 Nuffield Department of Surgical Sciences, University of Oxford, Oxford OX3 9DU, UK; newton.howard@nds.ox.ac.uk; 10 D-Lab, Massachusetts Institute of Technology, Cambridge, MA 02139, USA; fletcher@media.mit.edu; 11 Department of Psychiatry, University of Massachusetts Medical School, Worcester, MA 01655, USA; 12 School of Electrical and Computer Engineering, University of British Columbia, Vancouver, BC V6T 1Z4, Canada; rababw@ece.ubc.ca; 13 BC Children’s & Women’s Hospital, Vancouver, BC V6H 3N1, Canada

**Keywords:** photoplethysmogram, PPG signal, pulse oximetry, hypertension assessment, hypertension diagnosis, blood pressure measurement, wearable technology, wearable devices, digital health, digital medicine, pulse arrival time, biomedical engineering

## Abstract

Elevated blood pressure (BP) is a major cause of death, yet hypertension commonly goes undetected. Owing to its nature, it is typically asymptomatic until later in its progression when the vessel or organ structure has already been compromised. Therefore, noninvasive and continuous BP measurement methods are needed to ensure appropriate diagnosis and early management before hypertension leads to irreversible complications. Photoplethysmography (PPG) is a noninvasive technology with waveform morphologies similar to that of arterial BP waveforms, therefore attracting interest regarding its usability in BP estimation. In recent years, wearable devices incorporating PPG sensors have been proposed to improve the early diagnosis and management of hypertension. Additionally, the need for improved accuracy and convenience has led to the development of devices that incorporate multiple different biosignals with PPG. Through the addition of modalities such as an electrocardiogram, a final measure of the pulse wave velocity is derived, which has been proved to be inversely correlated to BP and to yield accurate estimations. This paper reviews and summarizes recent studies within the period 2010–2019 that combined PPG with other biosignals and offers perspectives on the strengths and weaknesses of current developments to guide future advancements in BP measurement. Our literature review reveals promising measurement accuracies and we comment on the effective combinations of modalities and success of this technology.

## 1. Introduction

Hypertension, or high blood pressure (BP), is a dangerous cardiac condition that accelerates end organ damage if not properly managed [1]. As this condition does not present noticeable symptoms early in its course, it is often not revealed unless regular BP monitoring is performed by health professionals or through self-management. Therefore, BP monitoring is a vital component of the diagnosis, management, and treatment of hypertension and early intervention is key for preventing its negative outcomes [2]. Recent years have seen considerable interest in developing a technology that is capable of accurately measuring continuous BP pressure both noninvasively and conveniently. Conventional cuff-based BP measurement methods are unable to measure BP continuously and are inconvenient and interruptive when measuring the ambulatory BP throughout the day and night. 

In multimodal biosignal systems, the most common feature used to estimate BP is the pulse wave velocity (PWV) [3]. The PWV is the speed at which a pressure wave travels down a segment of fixed length *d* and it can be calculated as PWV = *d*/PTT, where PTT is the pulse transit time. Based on the same idea, the pulse arrival time (PAT) is also tested and examined, and both PTT and PAT have been found to be closely correlated with BP [4]. The PTT and PAT durations reflect the time required for an arterial pressure wave to travel between a proximal and a distal site of an artery [5]. The difference being that the PAT includes an initial measurement at the heart, commonly measured using electrocardiography (ECG), whereas the initial measurement site for PTT is located distally, calculated by multi-site photoplethysmography (PPG) [6] or through utilization of other biosignals. PAT and PTT measurements both require the accurate detection of pulse at the distal artery. This has most commonly been measured using PPG as it has the ability to detect changes in blood pulsation through monitoring the variation in light absorption from a photodetector placed near a light-emitting diode [7]. Further details regarding the extraction of specific feature(s) from a single PPG signal and how these features can be incorporated into BP estimations are discussed and examined in our previous work [4,6,8,9,10]. This review focuses on evaluating various PPG-based multimodal biosignal systems that have been investigated by researchers in an attempt to estimate BP and assess hypertension. 

### Combining Other Biosignals with PPG for BP Estimation

**Figure 1:**
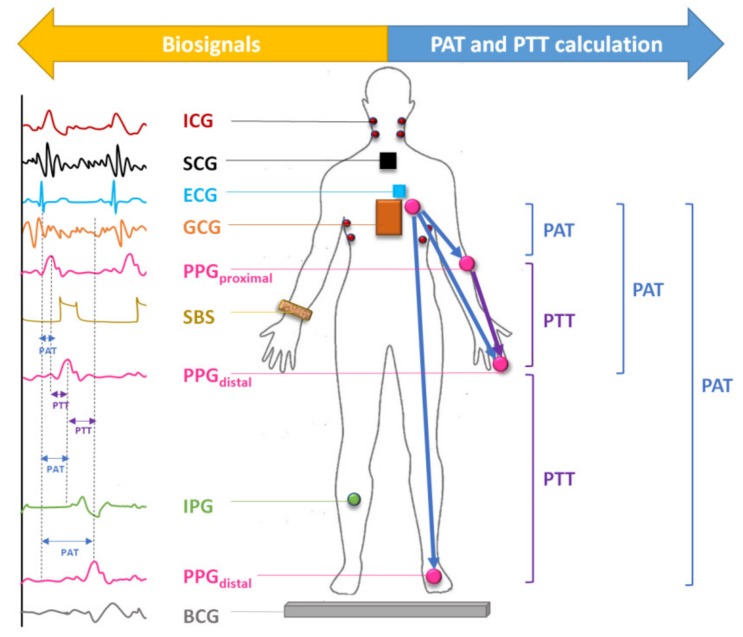
Determination of pulse arrival time and pulse transit time in various modalities that combine photoplethysmography with biosignals for blood pressure estimation. ICG: impedance cardiography, SCG: seismocardiography, ECG: electrocardiography, GCG: gyrocardiography, IPG: impedance plethysmography, PPG: photoplethysmography, BCG: ballistocardiography, SBS: strain-based sensor, PAT: pulse arrival time, PTT: pulse transit time.

In recent literature, various photoplethysmography-based modalities have been introduced to improve the assessment of hypertension, as shown in Figure 1. One of these modalities is the simultaneous collection of electrocardiography (ECG) with PPG to estimate PAT [4]. The PAT is defined as the time required for an arterial pressure wave to travel from the aorta to a peripheral location, beginning from the electrical activation of the heart [11]. In practice, it is calculated based on the time difference between the proximal QRS complex in the ECG waveform and the peak of the first derivative of the PPG waveform measured at a distal artery in the same cardiac cycle [12]. It is important to note that when PAT is measured from the Q-wave in ECG, it includes the pre-ejection period (PEP), which is defined as the electromechanical delay and period of isovolumetric contraction in the left ventricle [13]. PEP has been shown to account for 12%–35% of PAT [14] and to significantly skew BP estimations when not accounted for [13]. To derive the most accurate BP measurement from PAT, it is suggested that the PEP be measured and removed, resulting in a measure of PTT [13].

To account for PEP, several studies have explored other technologies to derive a more accurate representation of the opening of the aortic valve (AO) for use as a proximal timing indicator for PTT, therefore removing the contribution of PEP. Wong et al. [13] successfully used impedance cardiography (ICG) to calculate PEP based on the time difference between the ECG-Q-point and the ICG-B point and removed it to yield a more accurate measurement of PTT. Kim et al. [15] investigated the use of ballistocardiography (BCG) as a replacement for ECG as it does not include the PEP when measured from the BCG-J-wave and can therefore potentially yield a more precise proximal timing reference for PTT. Martin et al. [5] later confirmed the accuracy of this method by investigating a bathroom-scale-like device that used BCG and foot PPG waveforms to successfully track BP better than PAT-BP [16].

Seismocardiography (SCG) measures the thoracic vibrations of the heart through an accelerometer on the chest and the AO can be extracted from SCG waveforms, therefore avoiding the influence of PEP [17]. However, this modality is sensitive to movement artifacts and interindividual variability in morphology. Zhang et al. [11] proposed a wearable device and a supervised machine learning algorithm to help correct for this limitation that proved to increase the BP measurement accuracy. Similar to SCG, gyrocardiography (GCG) uses a gyroscope on the sternum to identify the AO by locating the maximum on the GCG wave in each cardiac cycle. Yang et al. [18] extracted the AO from SCG and GCG signals from the accelerometer and gyroscope of a smartphone, respectively, and combined this with the PPG signals measured using a modified optical sensor in the audio output to calculate PTT. The smartphone device resulted in superior PTT measurements compared to the reference PTT calculated with standalone sensors.

Other less common biosignals have also been investigated over the years to determine their suitability for incorporation into these modalities. For example, impedance plethysmography (IPG), which is defined as the electrical impedance of body tissues to an electric current, can be used to measure the amount of blood in blood vessels. Ibrahim et al. [19] calculated PTT using the time difference between the point of maximum negative inclination on the IPG waveform and the characteristic point on the PPG waveform. Ibrahim et al.’s study [19], along with one conducted by Huynh et al. [20], incorporated ICG sensors at the wrist as the proximal timing reference to a PPG sensor at the finger to yield a measurement of PTT that was more conveniently detected and comfortable for the user than the traditional ECG-PPG modality. The ICG-PPG modality produced correlation coefficients of 0.84 [19] and 0.88 [20] for SBP estimation, however very small sample sizes of *n* = 3 [19] and *n* = 15 [20] indicates the need for further validation. Overall, these results suggest that incorporating ICG into PTT based BP estimation techniques holds potential for yielding accurate results with a wearable and user-friendly device. 

This review aims to summarize recent technological advancements and offer perspectives on recent literature concerning the combination of PPG and other biosignal modalities for continuous, noninvasive BP measurement. In our analysis of scientific publications from January 2010 to January 2019, we highlight several factors that are important for future research, including global trends, the comparison to a validated gold standard, and appropriate subject recruitment. We summarize the overall accuracy reported in the included studies and a breakdown by the used modality combinations to provide recommendations and direction for future research endeavors.

## 2. Methods

### 2.1. Literature Search

**Figure 2:**
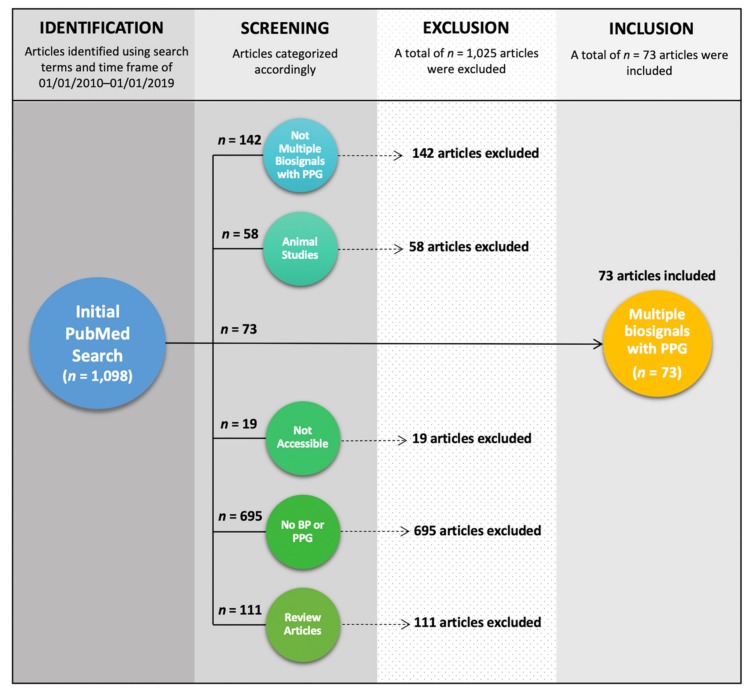
Flowchart of the methodology used to include 73 out of 1,098 published studies from 2010–2019.

We used the PubMed database to search for literature that investigates a combination of PPG technology and other biosignals for BP measurement. As we were interested in the most recent technological advancements, we chose to include studies from the most recent decade; studies were published between the dates 1 January 2010 and 1 January 2019. The search terms we used were a combination of the Medical Subject Heading terms “blood pressure,” “blood pressure determination,” “arterial pressure,” “hypertension,” “photoplethysmography,” and “pulse wave analysis,” as well as the general terms “analysis,” “classification,” “blood pressure monitoring,” “pulse wave analysis,” and “pulse transit time.” We chose to exclude “arterial stiffness” by including the search term “NOT arterial stiffness,” as the inclusion of this term yielded many studies that investigated the impact of vascular disease on biosignals but were not directly related to BP estimation. In total, the search yielded 1,098 results; 73 of which met our inclusion criteria and were included in the analysis. Papers were included in our review if they could be accessed in English and used PPG signals with simultaneously recorded but different biosignals to obtain BP estimations. We excluded review articles, animal studies, and studies that did not use both PPG and a different modality to calculate BP (see Figure 2). We recognize that a potential limitation of our review is the possibility of search engine bias. Only articles that were tagged with these terms were included and therefore, qualifying articles may have been missed in the search.

### 2.2. Statistical Analysis

As part of our analysis, we decided to present two of the most consistently reported measurements of accuracy to summarize the outcomes in the field. The mean error and standard deviation were extracted from studies that compared the estimated and reference BP for both SBP and DBP. Of the 73 included studies, 14 reported these values. Studies that did not include the BP mean error and standard deviation data were excluded from this error analysis. The second value we collected was Pearson’s correlation efficient (*r*) as it was reported in several studies. Values were included if the *r* values were used to compare the estimated and reference BP for both SBP and DBP. We felt that this was an important inclusion criterion as accurately measuring DBP using PPG-based technologies remains challenging and is an area for improvement. Of the 73 studies that reported correlation results, 22 met this criterion. Three studies [21,22,23] included in this analysis reported the coefficient of determination (*r*^2^) instead of *r*. To include these studies, we used the square root (*r*) of this value. On a few occasions, studies did not report an overall mean error or *r* and instead reported multiple values of each population they tested. These studies separately analyzed male and female groups [21], healthy participants and participants with heart disease [22], and participants whose BP was estimated during static exercise and those whose BP was estimated with a cold pressor test [23]. In the first two, to account for the multiple *r* values and include them in our results, we took the average between the two study populations to produce a combined *r*. In the third, we used static exercise data on the grounds that it was more applicable to the results of this review. In addition, several studies reported correlations between PTT/PAT and BP or mean arterial pressure (MAP) instead of SBP and DBP. They were not included in this analysis in an attempt to standardize the extracted data.

## 3. Results

### 3.1. Global Distribution and Combinations of Biosignals Used in Literature

**Figure 3:**
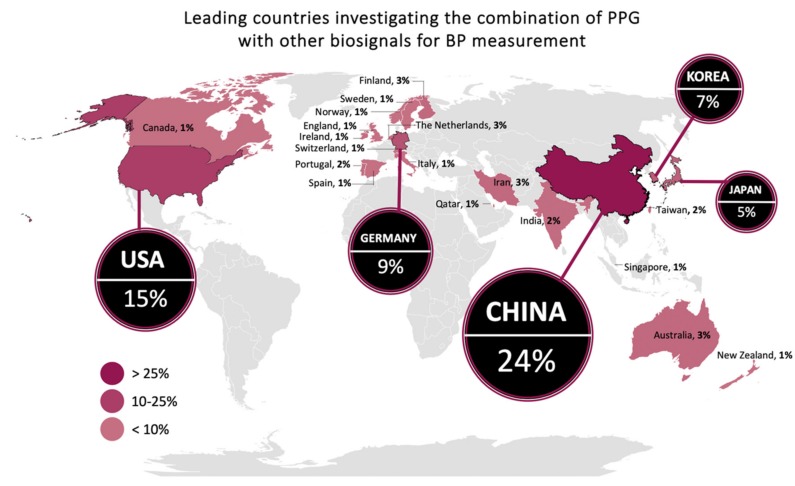
Global distribution of scientific articles that discussed PPG signals in combination with other biosignals to assess blood pressure from 2010–2019. The top five contributing countries are China (22), USA (14), Germany (8), Korea (6), and Japan (5), which collectively produced 67% of the publications. The number of publications per country is indicated by the intensity of the color, with darker colors representing a higher number of articles than lighter colors.

Our literature search returned 73 scientific publications that met our inclusion criteria, all of which were included in our analysis (Table 1). To gain a better understanding of the trends and geographical distribution of the included papers, we analyzed the data by year and country as well as the modalities used to calculate BP. The distribution by continent indicated that Asia provided the most significant contribution to the field with 42 publications, as shown in Figure 3. With Asia as the front runner in terms of volume of research, we recommend that other countries continue or increase their investigations in this field, given the substantial impact of hypertension on the health status and quality of life of individuals worldwide. As the intention of this research is to create a validated BP estimation device for use across various populations, the ideal device would be validated across individuals of various ethnic backgrounds from around the world.

**Figure 4:**
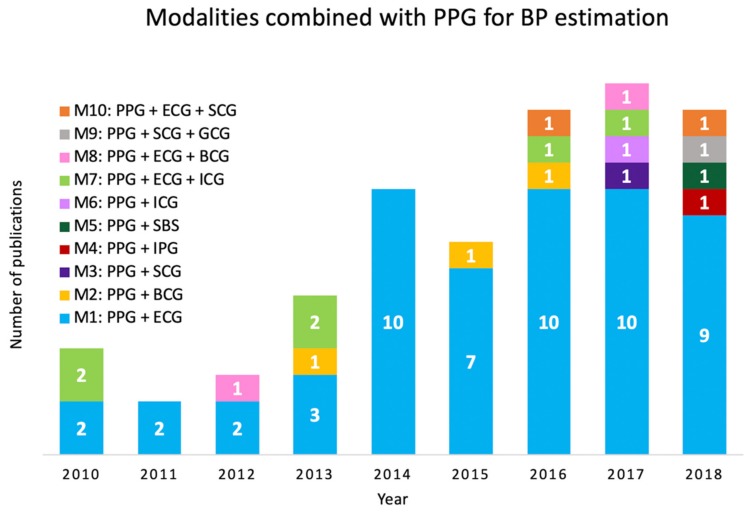
Number of publications per year and associated biosignal modalities that were combined with PPG from 2010–2019. M1-10: Various modality combinations of PPG: photoplethysmography with; ECG: electrocardiogram, SCG: seismocardiogram, BCG: ballistocardiogram, ICG: impedance cardiogram, GCG: gyrocardiogram, IPG: impedance plethysmography, and SBS: strain-based sensor. It can be observed that ECG is the dominant modality across all years.

Over the period January 2010–January 2019, there was an overall positive trend in the number of papers published each year that used multiple biosignals for PPG-BP measurement. In more recent years, we see a rising trend in the emergence of new combinations of various biosignals (Figure 4). 

Of the 73 included studies, 22 reported the correlation (*r*) between estimated and reference BP for both SBP and DBP [15,17,20,21,23,25,27,29,31,40,43,44,51,52,55,59,60,61,65,67,81,82]. Further, 15 studies reported the measurement variance in estimated versus reference BP for both SBP and DBP through mean error and standard deviation [21,22,33,34,38,51,59,60,64,67,69,72,78,82,83]. Various different biosignal combinations of ECG, BCG, SCG, ICG, IPG, and SBS were combined with PPG for BP estimation. Several studies investigated the possibility of including additional features, such PPG intensity ratio (PIR) and heart-power spectrum ratio (HRPS), into regression models in an attempt to improve accuracy, however their results were comparable to those of other studies. 

To determine the relative accuracy of the proposed technologies, we included the reported mean error and standard deviation (Table 1) as outlined in our methods. Overall, the studies that reported these values came exclusively from M1 (PPG + ECG), and the average mean error of studies that reported values for both SBP and DBP was 1.7 mmHg (SBP) and 1.3 mmHg (DBP).

**Figure 5:**
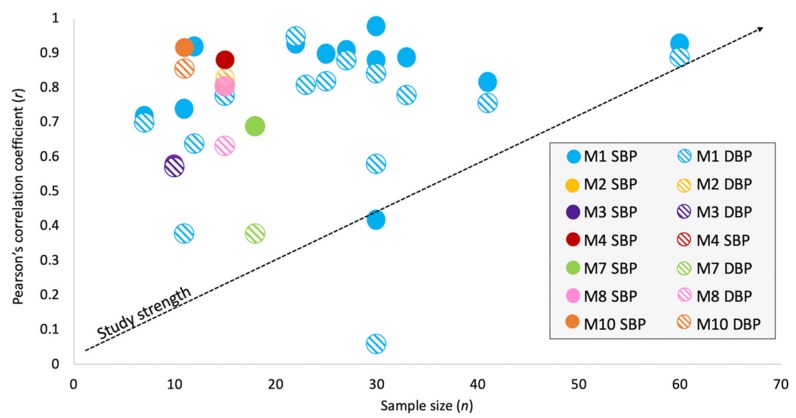
Relationship between reported correlation coefficients (*r*) of estimated vs. reference SBP and DBP and sample size (*n*) across all biosignal modalities. M1: modality 1 (PPG + ECG), M2: modality 2 (PPG + BCG), M3: modality 3 (PPG + SCG), M4: modality 4 (PPG + IPG), M7: modality 7 (PPG + ECG + ICG), M8: modality 8 (PPG + ECG + BCG), M10: modality 10 (PPG + ECG + SCG). Study strength (represented by the dashed line): the positive relationship between *r* and *n* in each study. If a study is able to achieve a high correlation over a large group of participants, it is less likely to be due to chance and it can be concluded that the correlation values are more likely to be true and validated over a variety of individuals. Therefore, data points are plotted by the strength of the correlation to the sample size and therefore, data points in the top right corner are considered to be the strongest, whereas those in the bottom left corner are considered to be the weakest. M1 is the modality with the most data points and the strongest *r*. M1 is also the only category to include studies with sample sizes over 30, making the correlations stronger than those of studies with small sample sizes. Across most modalities, SBP estimations have consistently higher correlations with real SBP than DBP estimations do with real DBP [17,21,23,25,27,40,43,44,51,52,55,60,61,65,81,82]. Three studies from M2 [15], M1 [59], and M1 [67] showed the opposite trend and two studies from M1 [31] and M4 [20] reported the same *r* for both SBP and DBP. Note that M5 (modality 5: PPG + SBS), M6 (modality 6: PPG + ICG), and M9 (modality 9: PPG + SCG + GCG) are not included in the above figure as these studies did not report correlation values for SBP and DBP.

Figure 5 shows the reported *r* from studies that included these values for both SBP and DBP. The validation of the correlation is compared to the sample size (*n*): the stronger the correlation and the larger the sample size, the more accurate the reported *r* may be. The average *r* for SBP and DBP, respectively, is 0.84 and 0.70 for M1, 0.81 and 0.81 for M2, 0.58 and 0.57 for M3, 0.88 and 0.88 for M4, 0.69 and 0.38 for M7, 0.81 and 0.63 for M8, and 0.92 and 0.85 for M10. Of the 55 studies in M1, the average *r* was calculated from the 15 studies that met this criterion, with the highest reported *r* for SBP and DBP being 0.98 and 0.84, respectively, and the lowest being 0.42 and 0.06, respectively. As only one study from each of M2, M3, M4, M7, M8, and M10 was included in this analysis of reported correlation, the average *r* reflects these single respective studies. Several studies did not report the correlation coefficients of their data and therefore, M5, M6, and M9 are not included in this analysis. It should be noted that this representation of accuracy does not reflect the entire dataset of included studies as measurements of accuracy were reported in different ways.

### 3.2. Sample Size and Patient Demographics

**Figure 6:**
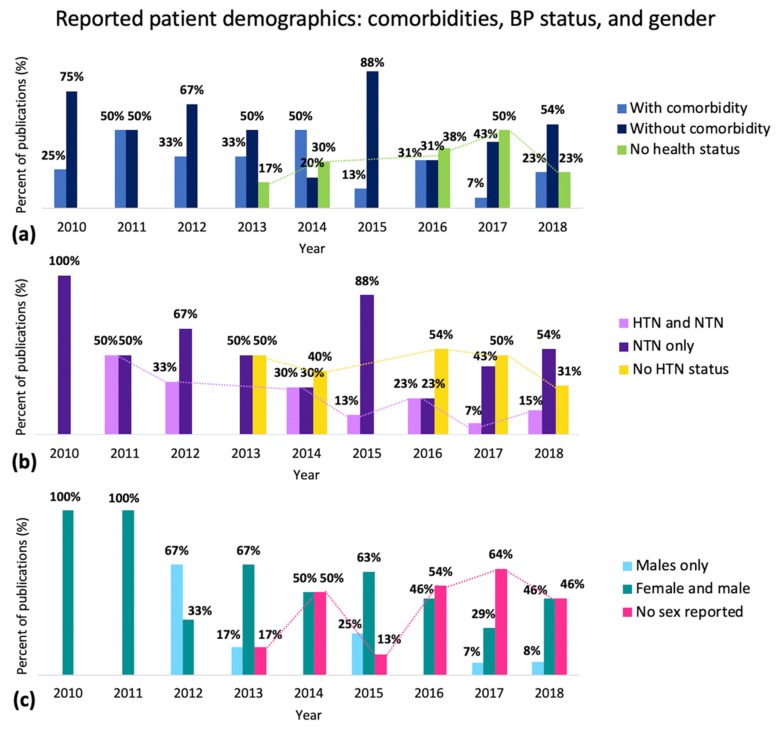
Patient demographics regarding health status, BP status, and gender reported in studies published from 2010–2019. (**a**) With comorbidity: reported diagnosed diseases in their participants. Without comorbidity: tested only healthy participants. No comorbidity reported: it was not reported if the subjects were normotensive vs. hypertensive and if the subjects had comorbidities or not. (**b**) HTN and NTN: documented number of subjects with and without hypertension. NTN only: only included healthy subjects. No HTN Status: did not disclose the BP status of their subjects. (**c**) Males only: tested only males. Female and male: tested both females and males. No gender reported: did not report the gender of their subjects.

The 17 studies with large sample sizes (*n* > 100) pulled subject data from databases; most commonly, they used the Medical Information Mart for Intensive Care (MIMIC) database. However, pulling data from this database is associated with a few notable limitations. As mentioned by Liang et al. [80], signals are assumed to be synchronized when collected simultaneously; however, this is not always the case. As some features are synchronicity-dependent, this can lead to unaccounted errors. In addition, patient data in this database are recorded from the ICU, which means that medications and comorbidities are most likely present; however, they are not included in the extracted data and are therefore not reported. Figure 6a–c shows a summary of the results in terms of comorbidities, BP, and gender.

In Figure 6a, the green bars show that there is a trend to not properly report the comorbidities of participants. In Figure 6b, the purple bars and yellow bars show that there is a declining trend with including hypertensive subjects in the studies. In Figure 6c, the dark pink bars show an increasing trend in not reporting the gender in their studies.

The most commonly reported comorbidities were hypertension, type 2 diabetes mellitus, cardiovascular disease, congestive heart failure, pulmonary disease, atrial fibrillation, and obstructive sleep apnea, all of which affect the cardiovascular system. Only 12 papers included participants with hypertension and 25 did not disclose BP status. Another interesting observation concerns the reporting of medications that coincide with subjects’ comorbidities. Out of the 19 studies that reported comorbidities, only five reported corresponding medications taken by their subject population. Some of the most commonly reported medications included beta blockers, calcium channel blockers, ACE inhibitors, diuretics, and nitrates. Three of the included studies [35,71,84] included intentionally administered medications as part of the experimental procedure. The majority of studies (i.e., 37) reported subjects of both genders. Surprisingly, 29 papers did not report the gender of their participants and seven studies included only men. No studies included pregnant women as participants.

### 3.3. Appropriate Gold Standard

Using an established gold standard to validate novel methods is an important factor affecting the quality of the evidence produced by these studies. Invasive arterial blood pressure (ABP) measurement through an arterial catheter and cuff-based arterial blood pressure (CBP) measurement with a sphygmomanometer are known to be the gold standards for BP measurement [85]. After analysis, we found that 11 studies were able to compare their BP estimations to ABP and 42 studies used CBP as their reference. As ABP is usually not feasible, owing to its invasive nature, many studies that used this measure extracted data from the MIMIC database or used patients who were under anesthesia in surgical conditions. Our analysis found that 17 studies used devices such as the Finometer, which uses PPG technology and the volume-clamp method to measure the finger arterial blood pressure (FABP), to obtain their reference BP measurements. The comparison to a non-validated device is noted as a limitation in the majority of these papers. Three studies did not involve a comparison with any of the above devices and instead directly compared their results to a PTT calculation from a previously investigated device.

## 4. Discussion

Our review provides readers and researchers with a relative summary of the technological advancements in the field of PPG and biosignals for BP measurement over recent years. Through our analysis, we discovered several noteworthy points related to the accuracy of the proposed methods, reporting of patient demographics, and testing conditions.

### 4.1. Accuracy of Proposed Methods

Most studies used ECG, which has been validated, owing to its consistently high correlations and low error. Of the studies included in our analysis of error, only seven met the criteria for validation based on the clinical standards set by the Association for the Advancement of Medical Instrumentation (AAMI), as seen in Table 1. In an attempt to improve accuracy, some researchers have incorporated HR into their calculations. For example, Wang et al. [38] showed a significant correlation between HR and BP and demonstrated that the addition of both HR and previous BP measurements to the PTT-BP estimation significantly improved the error (*p* < 0.05). Other measurements such as PIR have been incorporated into the PTT-BP estimation [16,51,67,69,71,78,82] as it reflects the low-frequency component of arterial diameter changes, which are not normally picked up by conventional PTT measurements. Here, PIR is measured as the ratio of minimum to maximum amplitudes of a PPG waveform in one cardiac cycle. Ding et al. [69] proved that the addition of PIR improved BP estimation, decreased error, and maintained accuracy over extended (24 h) calibration periods; however, other studies incorporating PIR struggled to obtain acceptable SD values. Recently, the heart-rate power spectrum ratio (HPSR), a new parameter, has been added to PTT-PIR estimation to more accurately identify the increase in sympathetic activity in hypertensive patients [82]. Alterations in sympathetic tone, adrenergic overflow, and parasympathetic tone contribute to changes in the factors that regulate BP and decrease the accuracy of PPT-BP estimation in this population. Chen et al. [82] concluded that the addition of HPSR resulted in superior estimation error, especially in the hypertensive population; however, the standard deviation was still higher than the acceptable standard set by the AAMI. It can be concluded that more work is needed surrounding decreasing error and standard deviation values before many of these proposed technologies meet the appropriate validation standards to be used clinically. 

The use of devices with SCG, BCG, and ICG has not been widely validated or accepted, largely owing to their susceptibility to error caused by motion artifacts and device complexity with an extra sensor [33,61]. Interestingly, one of the highest correlations (0.92 and 0.85 for SBP and DBP, respectively) was reported by a study [23] that investigated a combination of PPG, ECG, and SCG during static exercise; however, this method has not yet been validated during ambulation or for individuals other than young healthy males. To reduce the number of required measurement devices, researchers have become increasingly creative in trying to eliminate the need for ECG and produce a more compact design. For example, Huynh et al. [20] demonstrated that replacing ECG with IPG as a proximal timing reference for PTT can enhance BP estimation, reporting an *r* of 0.88 for both SBP and DBP. The two sensors were placed close to each other on the wrist and finger, indicating their potential application in a convenient wearable device. The results are limited by a small sample size (*n* = 15) and lack of hypertensive individuals.

Combining PPG with other biosignals for assessing comes with certain limitations, such as:

1. *Synchronization between biosignals*. If biosignals are not collected at the same time, PTT cannot be determined accurately. Unfortunately, this point is overlooked and therefore, there is inconsistency in reporting accuracy using PAT [86].
2. *Calibration.* Current PTT-BP estimations require calibration of PTT (ms) to BP (mmHg), which is dependent on several patient-specific factors: distance between measurement sites, average cross-sectional area of arteries between measurement sites, blood density, and the compliance of arteries [87]. As the composition of arteries changes with age (elastin in central arteries is replaced with collagen, which contributes to the atherosclerotic processes and stiffening of arteries), these techniques require subject specific calibration using a BP cuff at intervals in-line with atherosclerotic aging processes [87]. Mukkamala and Hahn [88] identified the maximum calibration interval to be about one year at age 30 with a linear decline to roughly six months by age 70. The need for calibration greatly limits the feasibility of these technologies, however new techniques such as those from Kachuee et al. [59] have eliminated the need for calibration using a machine learning approach. To avoid the calibration step (i.e., the step taken to map feature(s) values to mmHg), risk stratification could be used as an alternative output. In other words, build a model to classify PPG-based features into three classes: normotensive, pre-hypertensive, and hypertensive, rather than proving a specific mmHg value [9,10,80,89].
3. *Noise*. Biosignals are easily affected by noise, which can change the wave morphologies. To reduce noise in PPG signals, three main approaches were proposed in the literature: the use of filtering [90,91], signal quality index [92], and machine learning [73].

These challenges that impact accuracy need to be addressed properly before running a study and reporting findings. 

### 4.2. Reporting of Patient Demographics

Our analysis of patient demographics showed that most published papers investigating the use of PPG with other biosignals for BP estimation include a study population of healthy, normotensive individuals with no reported comorbidities. As BP monitoring devices will inherently be used more often by people that have hypertension, researchers must be careful and thorough in collecting data from this important population. Kim et al. [31] stated that the PTT-BP relationship in patients with chronic heart failure is inconsistent and worsens with disease progression. This suggests that the modified transmission of pulse waves in arteries experiencing disease-induced structural and functional changes is a potential confounding factor. The finding of decreased PTT-BP accuracy in patients with heart disease was also confirmed by Spiesshofer et al. [32] and Ding et al. [22].

### 4.3. Testing Conditions

In addition to reporting patient health status, we recommend that studies also report the corresponding medications that participants are taking for their comorbidities. Given that a large proportion of the population manages health conditions with medications, it is important to evaluate the contribution that they have on the accuracy of PTT-based BP estimations. It has been shown that vasoactive drugs significantly affect the calibration between PTT and mean arterial BP, and recalibration is necessary to mitigate these changes and maintain measurement accuracy [41]. Additionally, the administration of vasoactive drugs has been found to alter the vascular smooth muscle tone and, in turn, the reliability of PTT for adequately predicting BP [35,71,84]. Therefore, it is imperative that medications be reported and results be interpreted with this consideration in mind. Additionally, PPT-BP measurements have been evaluated for use in patients undergoing general anesthesia [25,41] as the induction of general anesthesia has been noted to be associated with considerable hemodynamic instability in hypertensive patients. Kim et al. [31] investigated whether PTT-based BP measurements were applicable in these conditions and concluded that such measurements could successfully track the associated SBP changes.

### 4.4. Recommendations for Future Advancements

The current literature on BP measurement using multiple biosignals and PPG is promising. Our key recommendations for future research endeavors are as follows:

− implementation of the IEEE guidelines for research regarding cuff-less BP devices, which includes information pertaining to observer training and measurement, validation procedures and appropriate gold standards, validation criteria, subject recruitment, and data reporting [93];
− inclusion of hypertensive and pregnant populations with improved reporting of gender and health status;
− appropriate definition and application of PAT and PTT that account for PEP to improve the measurement accuracy and decrease error;
− continued research on wearable devices that tests subjects during various ambulation activities, especially research on noise- and calibration-reducing algorithms.

## 5. Conclusions

Hypertension is one of the most important risk factors for cardiovascular diseases and is the leading cause of death worldwide. Thus, accurate BP monitoring is vital for effective management. Therefore, the development of a noninvasive device for continuous BP measurement would afford significant advantages to the medical community. However, research on hypertensive and pregnant individuals is currently lacking and more research is needed to understand how these variations contribute to the validity of results in these populations. Studies have tried to develop increasingly compact yet accurate devices over recent years; however, current technologies face limitations in successfully incorporating PPG and ECG into a single wearable device. Nonetheless, the successful validation of a publicly available wearable device has the potential to increase awareness and self-management strategies for the improved global prevention and management of cardiovascular disease.

## Figures and Tables

**Table 1:** Summary findings of the 73 papers included in this review. Studies that reported values for multiple study populations have been averaged for simplicity as per our methods and represented in italic. Studies that achieved mean difference and standard deviation for both SBP and DBP within the ANSI/AAMI/ISO 81060–2:2013 (mean difference of test versus reference BP measurements ≤ 5 mmHg with standard deviation ≤ 8 mmHg for systolic and diastolic BP) [24] are represented in bold. M: males, F: females, BP: blood pressure, NTN: normotensive, HTN: hypertensive, ABP: invasive arterial blood pressure, CBP: cuff blood pressure, FABP: finger arterial blood pressure, PAT: Pulse arrival time, PTT: Pulse transit time, ME: mean error, SD: standard deviation, SBP: systolic blood pressure, DBP: diastolic blood pressure, N/R: not reported, ECG: electrocardiogram, SCG: seismocardiogram, BCG: ballistocardiogram, ICG: impedance cardiogram, GCG: gyrocardiogram, IPG: impedance plethysmography, SBS: strain-based sensor, PIR: PPG intensity ratio, HPSR: heart-power spectrum ratio. M1: modality 1 (PPG + ECG), M2: modality 2 (PPG + BCG), M3: modality 3 (PPG + SCG), M4: modality 4 (PPG + IPG), M5: modality 5 (PPG + SBS), M6: modality 6 (PPG + ICG), M7: modality 7 (PPG + ECG + ICG), M8: modality 8 (PPG + ECG + BCG), M9: modality 9 (PPG + SCG + GCG), M10: modality 10 (PPG + ECG + SCG).

Publication	# Subjects (M:F)	BP Status	Comorbidities	Gold Standard	Modality Category	ME ± SD (mmHg)	Pearson’s Coefficient (*r)*
Baek et al. (2010) [25]	15 (11:4)	NTN	Yes	ABP, FABP	M1	SBP = N/R DBP = N/R	SBP = 0.815 DBP = 0.779
Chua et al. (2010) [26]	18 (14:4)	NTN	No	FABP	M1	SBP = N/R DBP = N/R	SBP = 0.73 DBP = N/R
Proença et al. (2010) [12]	20 (14:6)	NTN	No	FABP	M7	SBP = N/R DBP = N/R	SBP = N/R DBP = N/R
Wong et al. (2011) [13]	22 (14:8)	NTN	No	ABP	M7	SBP = N/R DBP = N/R	SBP = N/R DBP = N/R
Mase et al. (2011) [27]	33 (19:14)	NTN, HTN	Yes	CBP	MI	SBP = N/R DBP = N/R	SBP = 0.89 DBP = 0.78
Gesche et al. (2012) [28]	63 (36:27)	NTN	No	CBP	M1	SBP = N/R DBP = N/R	SBP = 0.83 DBP = N/R
Kato et al. (2012) [29]	1 (1:0)	NTN	No	CBP	M8	SBP = N/R DBP = N/R	SBP = 0.805 DBP = 0.633
Baek et al. (2012) [30]	5 (5:0)	NTN	No	FABP	M1	SBP = N/R DBP = N/R	SBP = 0.848 DBP = N/R
Kim et al. (2013) [31]	23 (17:6)	HTN	Yes	ABP	M1	SBP = N/R DBP = N/R	SBP = 0.81 DBP = 0.81
Spießhöfer et al. (2013) [32]	29 (27:2)	N/R	Yes	CBP	M1	SBP = N/R DBP = N/R	SBP = N/R DBP = N/R
Chen et al. (2013) [33]	5 (N/R)	NTN	No	CBP	M2	SBP = 9.0 ± 5.6 DBP = 1.8 ± 1.3	SBP = N/R DBP = N/R
Puke et al. (2013) [34]	4 (3:1)	N/R	N/R	CBP	M1	SBP = 6.91 ± 4.23 DBP = N/R	SBP = N/R DBP = N/R
Couceiro et al. (2013) [35]	43 (23:20)	N/R	Yes	FABP	M7	SBP = N/R DBP = N/R	SBP = N/R DBP = N/R
Solà et al. (2013) [36]	15 (15:0)	NTN	No	CBP	M7	SBP = N/R DBP = N/R	SBP = N/R DBP = N/R
Jeong & Finkelstein (2013) [37]	5 (2:3)	NTN	No	CBP	M1	SBP = N/R DBP = N/R	SBP = N/R DBP = N/R
***Wang et al. (2014)*** **[38]**	***6 (N/R)***	***N/R***	***N/R***	***CBP***	***M1***	***SBP = 0.04 ± 3.78*** ***DBP = −0.01 ± 4.34***	***SBP = N/R*** ***DBP = N/R***
Thomas et al. (2014) [39]	4 (N/R)	N/R	N/R	CBP	M1	SBP = N/R DBP = N/R	SBP = N/R DBP = N/R
Ma, HT (2014) [40]	30 (N/R)	NTN	No	CBP	M1	SBP = N/R DBP = N/R	SBP R2 = 0.96 DBP R2 = 0.71
Zhang et al. (2014) [41]	2 (N/R)	N/R	Yes	ABP	M1	SBP = N/R DBP = N/R	SBP = N/R DBP = N/R
Vlahandonis et al. (2014) [42]	25 (12:18)	NTN	Yes	CBP	M1	SBP = N/R DBP = N/R	SBP = N/R DBP = N/R
*Younessi heravi et al. (2014)* [21]	*25 (15:10)*	*N/R*	*N/R*	*CBP*	*M1*	*SBP = 6.73* ± *2.68* *DBP = 8.13* ± *3.18*	*SBP = 0.89* *DBP = 0.82*
Gomez Garcia et al. (2014) [43]	30 (20:10)	NTN, HTN	Yes	CBP	M1	SBP = −0.2 ± 2.4 DBP = N/R	SBP = 0.88 DBP = 0.58
Wibmer et al. (2014) [44]	20 (14:6)	NTN, HTN	Yes	CBP	M1	SBP = N/R DBP = N/R	SBP R2 = 0.92 DBP R2 = 0.46
Zheng et al. (2014) [45]	10 (N/R)	NTN	No	CBP	M1	SBP = 2.8 ± 8.2 DBP = N/R	SBP = N/R DBP = N/R
Liu et al. (2014) [46]	46 (34:7)	NTN, HTN	Yes	CBP, FABP	M1	SBP = N/R DBP = N/R	SBP = N/R DBP = N/R
Liu et al. (2015) [47]	10 (6:4)	NTN	No	CBP	M1	SBP = N/R DBP = N/R	SBP = N/R DBP = N/R
Tang et al. (2015) [48]	9 (9:0)	NTN	No	CBP, FABP	M1	SBP = N/R DBP = N/R	SBP = N/R DBP = N/R
Ding & Zhang (2015) [16]	5 (N/R)	NTN	No	FABP	M1 + PIR	SBP = N/R DBP = N/R	SBP = N/R DBP = N/R
Tamura et al. (2015) [49]	9 (9:0)	NTN	No	FABP	M1	0.7 ± 3.65 (Unclear if SBP or DBP)	SBP = N/R DBP = N/R
Kim et al. (2015) [15]	15 (10:5)	NTN	No	PAT	M2	SBP = N/R DBP = N/R	SBP = 0.81 DBP = 0.83
Wibmer et al. (2015) [50]	18 (11:7)	NTN, HTN	Yes	CBP	M1	SBP = N/R DBP = N/R	SBP = 0.93 DBP = N/R
**Ding et al. (2016)** **[51]**	**27 (14:13)**	**NTN**	**No**	**FABP**	**M1 + PIR**	**SBP = −0.037 ± 5.21** **DBP = −0.08 ± 4.06**	**SBP = 0.91** **DBP = 0.88**
Thomas et al. (2016) [52]	11 (N/R)	NTN	No	CBP	M1	SBP = N/R DBP = N/R	SBP = 0.72 DBP = 0.70
Sun et al. (2016) [53]	19 (14:5)	N/R	No	FABP	M1	SBP = 0.43 ± 13.52 DBP = N/R	SBP = 0.93 DBP = N/R
*Ding et al. (2016)* [22]	*85 (37:48)*	*NTN*, *HTN*	*Yes*	*CBP*	*M1*	*SBP =* −*1.55* ± *13.79* *DBP = 0.07* ± *8.49*	*SBP = N/R* *DBP = N/R*
Martin et al. (2016) [5]	22 (19:3)	NTN	No	FABP	M2	SBP = N/R DBP = N/R	SBP = N/R DBP = N/R
Dai et al. (2016) [54]	7 (N/R)	NTN	No	FABP	M1	SBP = N/R DBP = N/R	SBP = N/R DBP = N/R
Zhang et al. (2016) [11]	2 (N/R)	NTN	N/R	CBP	M8	SBP = N/R DBP = N/R	SBP = N/R DBP = N/R
Shahrbabaki et al. (2016) [55]	10 (N/R)	N/R	N/R	CBP	M1	SBP = N/R DBP = N/R	SBP R2 = 0.59 DBP R2 = 0.42
Gholamhosseini et al. (2016) [56]	13 (N/R)	N/R	N/R	CBP	M1	SBP = N/R DBP = N/R	SBP = N/R DBP = N/R
Schoot et al. (2016) [57]	37 (18:19)	NTN, HTN	Yes	CBP	M1	SBP = N/R DBP = N/R	SBP = N/R DBP = N/R
Jain et al. (2016) [58]	72 (N/R)	N/R	N/R	CBP	M1	SBP = N/R DBP = N/R	SBP = N/R DBP = N/R
Liu et al. (2016) [7]	20 (N/R)	NTN, HTN	Yes	FABP	M1	SBP = N/R DBP = N/R	SBP R2 = 0.95 DBP = N/R
Kachuee et al. (2017) [59]	1000 (N/R)	N/R	N/R	ABP	M1	SBP = −0.06 ± 9.88 DBP = 0.36 ± 5.7	SBP = 0.54 DBP = 0.57
**Tang et al. (2017)** **[60]**	**12 (11:1)**	**NTN, HTN**	**No**	**ABP**	**M1**	**SBP = 0.2 ± 5.8** **DBP = 0.4 ± 5.7**	**SBP = 0.92** **DBP = 0.64**
*Seeberg et al. (2017)* [61]	*18 (15:3)*	*N/R*	*Yes*	*CBP*, *FABP*	*M7*	*SBP = N/R* *DBP = N/R*	*SBP = 0.69* *DBP = 0.38*
Janjua et al. (2017) [62]	11 (9:2)	NTN	No	CBP	M8	SBP = N/R DBP = N/R	SBP = N/R DBP = N/R
Liu et al. (2017) [63]	20 (N/R)	NTN	No	CBP	M7	SBP = N/R DBP = N/R	SBP = 0.7 DBP = N/R
**Chen et al. (2017)** **[64]**	**10 (5:5)**	**NTN**	**No**	**FABP**	**M1**	**SBP = −0.91 ± 3.84** **DBP = −0.36 ± 3.36**	**SBP = N/R** **DBP = N/R**
Ahmaniemi et al. (2017) [65]	30 (N/R)	NTN	No	CBP	M1	SBP = N/R DBP = N/R	SBP = 0.42 DBP = 0.06
Zhang et al. (2017) [66]	10 (7:3)	N/R	N/R	CBP	M1	SBP = 1.63 ± 4.4 DBP = N/R	SBP = N/R DBP = N/R
Lin et al. (2017) [67]	22 (N/R)	NTN	No	CBP	M1 + PIR	SBP = 3.22 ± 8.02 DBP = 3.13 ± 4.82	SBP = 0.93 DBP = 0.95
Bhattacharya et al. (2017) [68]	6 (N/R)	N/R	N/R	CBP	M1	SBP = N/R DBP = N/R	SBP = N/R DBP = N/R
**Ding et al. (2017)** **[69]**	**33 (N/R)**	**NTN, HTN**	**Yes**	**FABP**	**M1 + PIR**	**SBP = 1.17 ± 5.72** **DBP = 0.46 ± 5.49**	**SBP = N/R** **DBP = N/R**
Pflugradt et al. (2017) [70]	N/R (N/R)	N/R	N/R	ABP	M1	SBP = 0.015 ± 4.41 DBP = N/R	SBP = N/R DBP = N/R
Ding et al. (2017) [71]	6 (N/R)	N/R	N/R	ABP	M1 + PIR	SBP = N/R DBP = N/R	SBP = N/R DBP = N/R
**Xu et al. (2017)** **[72]**	**10 (8:2)**	**N/R**	**N/R**	**CBP**	**M1**	**SBP = 4.5 ± 6.13** **DBP = 3.4 ± 3.37**	**SBP = N/R** **DBP = N/R**
Ibrahim et al. (2017) [19]	3 (N/R)	N/R	N/R	PTT, FABP	M6	SBP = N/R DBP = N/R	SBP = 0.84 DBP = N/R
Lo et al. (2017) [73]	25 (N/R)	N/R	N/R	ABP	M1	SBP = N/R DBP = N/R	SBP = N/R DBP = N/R
Yang & Tavassolian (2018) [17]	10 (10:0)	NTN	No	CBP	M3	SBP = N/R DBP = N/R	SBP = 0.58 DBP = 0.57
Rajala et al. (2018) [74]	30 (19:11)	NTN	No	CBP	MI	SBP = N/R DBP = N/R	SBP = 0.37 DBP = N/R
Wang et al. (2018) [75]	59 (N/R)	N/R	N/R	CBP	M5	SBP = N/R DBP = N/R	SBP = N/R DBP = N/R
Lin et al. (2018) [76]	22 (N/R)	NTN	No	FABP	M1	SBP = N/R DBP = N/R	SBP = N/R DBP = N/R
Kim et al. (2018) [77]	N/R (N/R)	N/R	N/R	PAT	M1	SBP = N/R DBP = N/R	SBP = N/R DBP = N/R
*Sharifi et al. (2018)* [78]	*1000 (N/R)*	*N/R*	*N/R*	*ABP*	*M1 + PIR*	*SBP =* −*0.29* ± *9.1 * *DBP =* −*0.1* ± *8.62*	*SBP = N/R* *DBP = N/R*
Ahmaniemi et al. (2018) [79]	10 (9:1)	NTN	No	FABP	MI	SBP = 9.8 DBP = N/R	SBP = 0.75 DBP = N/R
Liang et al. (2018) [80]	121 (N/R)	NTN, HTN	Yes	ABP	M1	SBP = N/R DBP = N/R	SBP = N/R DBP = N/R
Yang et al. (2018) [18]	10 (N/R)	NTN	No	PTT	M9	SBP = N/R DBP = N/R	SBP = N/R DBP = N/R
Xu et al. (2018) [81]	41 (21:21)	NTN	No	CBP	M1	SBP = N/R DBP = N/R	SBP = 0.817 DBP = 0.757
Chen et al. (2018) [82]	60 (40:20)	NTN, HTN	Yes	CBP	M1 + PIR, HPSR	SBP = 0.61 ± 9.36 DBP = 0.68 ± 6.67	SBP = 0.93 DBP = 0.89
Lee et al. (2018) [23]	11 (11:0)	NTN	No	FABP	M10	SBP = N/R DBP = N/R	SBP = 0.915 DBP = 0.854
**Feng et al. (2018)** **[83]**	**28 (15:13)**	**N/R**	**Yes**	**ABP**	**M1**	**SBP = −0.98 ± 6.0** **DBP = 0.02 ± 4.98**	**SBP = N/R** **DBP = N/R**
Huynh et al. (2018) [20]	15 (10:5)	NTN	No	CBP	M4	SBP = N/R DBP = N/R	SBP = 0.88 DBP = 0.88

## Abbreviations

Abbreviations of terms used in this review.

BP	Blood pressure	M1	Modality 1 (PPG + ECG)
PPG	Photoplethysmography	M2	Modality 2 (PPG + BCG)
PTT	Pulse transit time	M3	Modality 3 (PPG + SCG)
PAT	Pulse arrival time	M4	Modality 4 (PPG + IPG)
PWV	Pulse wave velocity	M5	Modality 5 (PPG + SBS)
PEP	Pre-ejection period	M6	Modality 6 (PPG + ICG)
AO	Aortic opening	M7	Modality 7 (PPG + ECG + ICG)
ECG	Electrocardiography	M8	Modality 8 (PPG + ECG + BCG)
ICG	Impedance cardiography	M9	Modality 9 (PPG + SCG + GCG)
BCG	Ballistocardiography	M10	Modality 10 (PPG + ECG + SCG)
SCG	Seismocardiography	ME	Mean error
GCG	Gyrocardiography	SD	Standard deviation
IPG	Impedance plethysmography	MIMIC	Medical Information Mart for Intensive Care
SBS	Strain based sensor	AAMI	Association for the Advancement of Medical Instrumentation
MAP	Mean arterial pressure	HTN	Hypertension
SBP	Systolic blood pressure	NTN	Normotension
DBP	Diastolic blood pressure	ABP	Arterial invasive blood pressure
HR	Heart rate	CBP	Cuff blood pressure
PIR	PPG intensity ratio	FABP	Finger arterial blood pressure
HRPS	Heart-power spectrum ration	IEEE	Institute of Electrical and Electronics Engineers

## References

[1] Calvillo L., Gironacci M.M., Crotti L., Meroni P.L., Parati G. (2019). Neuroimmune crosstalk in the pathophysiology of hypertension. Nat. Rev. Cardiol..

[2] McCrindle B.W. (2010). Assessment and management of hypertension in children and adolescents. Nat. Rev. Cardiol..

[3] Nabeel P., Jayaraj J., Mohanasankar S. (2017). Single-source PPG-based local pulse wave velocity measurement: A potential cuffless blood pressure estimation technique. Physiol. Meas..

[4] Elgendi M., Fletcher R., Liang Y., Howard N., Lovell N.H., Abbott D., Lim K., Ward R. (2019). The use of photoplethysmography for assessing hypertension. NPJ Digit. Med..

[5] Martin S.L.O., Carek A.M., Kim C.-S., Ashouri H., Inan O.T., Hahn J.-O., Mukkamala R. (2016). Weighing Scale-Based Pulse Transit Time is a Superior Marker of Blood Pressure than Conventional Pulse Arrival Time. Sci. Rep..

[6] Chan G., Cooper R., Hosanee M., Welykholowa K., Kyriacou P.A., Zheng D., Allen J., Abbott D., Lovell N.H., Fletcher R. (2019). Multi-Site Photoplethysmography Technology for Blood Pressure Assessment: Challenges and Recommendations. J. Clin. Med..

[7] Liu J., Yan B.P., Dai W.X., Ding X.R., Zhang Y.T., Zhao N. (2016). Multi-wavelength photoplethysmography method for skin arterial pulse extraction. Biomed Opt. Express.

[8] Hosanee M., Chan G., Welykholowa K., Cooper R., Kyriacou P.A., Zheng D., Allen J., Abbott D., Menon C., Lovell N.H. (2020). Cuffless Single-Site Photoplethysmography for Blood Pressure Monitoring. J. Clin. Med..

[9] Martínez G., Howard N., Abbott D., Lim K., Ward R., Elgendi M. (2018). Can Photoplethysmography Replace Arterial Blood Pressure in the Assessment of Blood Pressure?. J. Clin. Med..

[10] Liang Y., Chen Z., Ward R., Elgendi M. (2018). Hypertension Assessment Using Photoplethysmography: A Risk Stratification Approach. J. Clin. Med..

[11] Zhang G., Cottrell A.C., Henry I.C., McCombie D.B. Assessment of pre-ejection period in ambulatory subjects using seismocardiogram in a wearable blood pressure monitor. Proceedings of the 38th Annual International Conference of the IEEE Engineering in Medicine and Biology Society (EMBC).

[12] Proença J., Muehlsteff J., Aubert X., Carvalho P. Is pulse transit time a good indicator of blood pressure changes during short physical exercise in a young population?. Proceedings of the 2010 Annual International Conference of the IEEE Engineering in Medicine and Biology.

[13] Wong M.Y., Pickwell-MacPherson E., Zhang Y.T., Cheng J.C. (2011). The effects of pre-ejection period on post-exercise systolic blood pressure estimation using the pulse arrival time technique. Eur. J. Appl. Physiol..

[14] Payne R., Symeonides C., Webb D., Maxwell S. (2006). Pulse transit time measured from the ECG: An unreliable marker of beat-to-beat blood pressure. J. Appl. Physiol..

[15] Kim C.S., Carek A.M., Mukkamala R., Inan O.T., Hahn J.O. (2015). Ballistocardiogram as Proximal Timing Reference for Pulse Transit Time Measurement: Potential for Cuffless Blood Pressure Monitoring. IEEE Trans. Biomed. Eng..

[16] Ding X.R., Zhang Y.T. Photoplethysmogram intensity ratio: A potential indicator for improving the accuracy of PTT-based cuffless blood pressure estimation. Proceedings of the Annual International Conference of the IEEE Engineering in Medicine and Biology Society.

[17] Yang C., Tavassolian N. (2018). Pulse transit time measurement using seismocardiogram, photoplethysmogram, and acoustic recordings: Evaluation and comparison. IEEE J. Biomed. Health Inform..

[18] Yang C., Dong Y., Chen Y., Tavassolian N. A Low-cost, Smartphone-only Pulse Transit Time Measurement System Using Cardio-mechanical Signals and Optical Sensors. Proceedings of the Annual International Conference of the IEEE Engineering in Medicine and Biology Society.

[19] Ibrahim B., Nathan V., Jafari R. Exploration and validation of alternate sensing methods for wearable continuous pulse transit time measurement using optical and bioimpedance modalities. Proceedings of the 39th Annual International Conference of the IEEE Engineering in Medicine and Biology Society (EMBC).

[20] Huynh T.H., Jafari R., Chung W.-Y. (2019). Noninvasive cuffless blood pressure estimation using pulse transit time and impedance plethysmography. IEEE Trans. Biomed. Eng..

[21] Younessi Heravi M.A., Khalilzadeh M.A., Joharinia S. (2014). Continuous and Cuffless Blood Pressure Monitoring Based on ECG and SpO2 Signals ByUsing Microsoft Visual C Sharp. J. Biomed. Phys. Eng..

[22] Ding X., Zhang Tsang H.K. (2016). Impact of heart disease and calibration interval on accuracy of pulse transit time-based blood pressure estimation. Physiol. Meas..

[23] Lee J., Sohn J., Park J., Yang S., Lee S., Kim H.C. (2018). Novel blood pressure and pulse pressure estimation based on pulse transit time and stroke volume approximation. Biomed. Eng. Online.

[24] Association for the Advancement of Medical Instrumentation (2013). International Standard: Non-Invasive Sphygmomanometers.

[25] Baek H.J., Kim K.K., Kim J.S., Lee B., Park K.S. (2010). Enhancing the estimation of blood pressure using pulse arrival time and two confounding factors. Physiol. Meas..

[26] Chua E.C.-P., Redmond S.J., McDarby G., Heneghan C. (2010). Towards Using Photo-Plethysmogram Amplitude to Measure Blood Pressure During Sleep. Ann. Biomed. Eng..

[27] Mase M., Mattei W., Cucino R., Faes L., Nollo G. (2011). Feasibility of cuff-free measurement of systolic and diastolic arterial blood pressure. J. Electrocardiol..

[28] Gesche H., Grosskurth D., Kuchler G., Patzak A. (2012). Continuous blood pressure measurement by using the pulse transit time: Comparison to a cuff-based method. Eur. J. Appl. Physiol..

[29] Kato Y., Nambu M., Imura M., Kuroda Y., Oshiro O. Smart sensing of cardiovascular physiological information from soles without direct skin contact. Proceedings of the Annual Conference of the IEE Engineering in Medicine and Biology Society.

[30] Baek H.J., Chung G.S., Kim K.K., Park K.S. (2012). A smart health monitoring chair for nonintrusive measurement of biological signals. IEEE Trans. Inf. Technol. Biomed..

[31] Kim S.H., Song J.G., Park J.H., Kim J.W., Park Y.S., Hwang G.S. (2013). Beat-to-beat tracking of systolic blood pressure using noninvasive pulse transit time during anesthesia induction in hypertensive patients. Anesth. Analg..

[32] Spießhöfer J., Heinrich J., Bitter T., Efken C., Lehmann R., Eckert S., Horstkotte D., Oldenburg O. (2013). Validation of blood pressure monitoring using pulse transit time in heart failure patients with Cheyne-Stokes respiration undergoing adaptive servoventilation therapy. Sleep Breath..

[33] Chen Z., Yang X., Teo J.T., Ng S.H. Noninvasive monitoring of blood pressure using optical Ballistocardiography and Photoplethysmograph approaches. Proceedings of the Annual International Conference of the IEE Engineering in Medicine and Biology Society.

[34] Puke S., Suzuki T., Nakayama K., Tanaka H., Minami S. Blood pressure estimation from pulse wave velocity measured on the chest. Proceedings of the Annual International Conference of the IEEE Engineering in Medicine and Biology Society.

[35] Couceiro R., Carvalho P., Paiva R.P., Muehlsteff J., Henriques J., Schulze V., Ritz A., Kelm M., Meyer C. Characterization of surrogate parameters for blood pressure regulation in neurally-mediated syncope. Proceedings of the Annual International Conference of the IEEE Engineering in Medicine and Biology Society.

[36] Sola J., Proenca M., Ferrario D., Porchet J.A., Falhi A., Grossenbacher O., Allemann Y., Rimoldi S.F., Sartori C. (2013). Noninvasive and Nonocclusive Blood Pressure Estimation Via a Chest Sensor. IEEE Trans. Biomed. Eng..

[37] Jeong I., Finkelstein J. (2013). Optimizing non-invasive blood pressure estimation using pulse transit time. Stud. Health Technol. Inform..

[38] Wang R., Jia W., Mao Z.-H., Sclabassi R.J., Sun M. Cuff-free blood pressure estimation using pulse transit time and heart rate. Proceedings of the 12th International Conference on Signal Processing (ICSP).

[39] Thomas S.S., Nathan V., Zong C., Akinbola E., Aroul A.L.P., Philipose L., Soundarapandian K., Shi X., Jafari R. BioWatch—A wrist watch based signal acquisition system for physiological signals including blood pressure. Proceedings of the 36th Annual International Conference of the IEEE Engineering in Medicine and Biology Society.

[40] Ma H.T. (2014). A blood pressure monitoring method for stroke management. BioMed Res. Int..

[41] Zhang G., McCombie S.A., Greenstein R., McCombie D.B. Assessing the challenges of a pulse wave velocity based blood pressure measurement in surgical patients. Proceedings of the Annual International Conference of the IEEE Engineering in Medicine and Biology Society.

[42] Vlahandonis A., Biggs S.N., Nixon G.M., Davey M.J., Walter L.M., Horne R.S. (2014). Pulse transit time as a surrogate measure of changes in systolic arterial pressure in children during sleep. J. Sleep Res..

[43] Gomez Garcia M.T., Troncoso Acevedo M.F., Rodriguez Guzman M., Alegre de Montaner R., Fernandez Fernandez B., del Rio Camacho G., Gonzalez-Mangado N. (2014). Can pulse transit time be useful for detecting hypertension in patients in a sleep unit?. Archivos De Bronconeumologia.

[44] Wibmer T., Doering K., Kropf-Sanchen C., Rudiger S., Blanta I., Stoiber K.M., Rottbauer W., Schumann C. (2014). Pulse transit time and blood pressure during cardiopulmonary exercise tests. Physiol. Res..

[45] Zheng Y.L., Yan B.P., Zhang Y.T., Poon C.C. (2014). An armband wearable device for overnight and cuff-less blood pressure measurement. IEEE Trans. Biomed. Eng..

[46] Liu Q., Yan B.P., Yu C.M., Zhang Y.T., Poon C.C. (2014). Attenuation of systolic blood pressure and pulse transit time hysteresis during exercise and recovery in cardiovascular patients. IEEE Trans. Biomed. Eng..

[47] Liu J., Li Y., Ding X.R., Dai W.X., Zhang Y.T. Effects of cuff inflation and deflation on pulse transit time measured from ECG and multi-wavelength PPG. Proceedings of the Annual International Conference of the IEEE Engineering in Medicine and Biology Society.

[48] Tang Z., Sekine M., Tamura T., Yoshida M., Chen W. A chair for cuffless real-time estimation of systolic blood pressure based on pulse transit time. Proceedings of the Annual International Conference of the IEEE Engineering in Medicine and Biology Society.

[49] Tamura T., Sekine M., Zunyi T., Yoshida M., Takeuchi Y., Imai M. Preliminary study of a new home healthcare monitoring to prevent the recurrence of stroke. Proceedings of the Annual International Conference of the IEEE Engineering in Medicine and Biology Society.

[50] Wibmer T., Denner C., Fischer C., Schildge B., Rudiger S., Kropf-Sanchen C., Rottbauer W., Schumann C. (2015). Blood pressure monitoring during exercise: Comparison of pulse transit time and volume clamp methods. Blood Press..

[51] Ding X.R., Zhang Y.T., Liu J., Dai W.X., Tsang H.K. (2016). Continuous Cuffless Blood Pressure Estimation Using Pulse Transit Time and Photoplethysmogram Intensity Ratio. IEEE Trans. Biomed. Eng..

[52] Thomas S.S., Nathan V., Zong C., Soundarapandian K., Shi X., Jafari R. (2016). BioWatch: A noninvasive wrist-based blood pressure monitor that incorporates training techniques for posture and subject variability. IEEE J. Biomed. Health Inform..

[53] Sun S., Bezemer R., Long X., Muehlsteff J., Aarts R. (2016). Systolic blood pressure estimation using PPG and ECG during physical exercise. Physiol. Meas..

[54] Dai W.-X., Zhang Y.-T., Liu J., Ding X.-R., Zhao N. Dual-modality arterial pulse monitoring system for continuous blood pressure measurement. Proceedings of the 38th Annual International Conference of the IEEE Engineering in Medicine and Biology Society (EMBC).

[55] Shahrbabaki S.S., Ahmed B., Penzel T., Cvetkovic D. Photoplethysmography derivatives and pulse transit time in overnight blood pressure monitoring. Proceedings of the 38th Annual International Conference of the IEEE Engineering in Medicine and Biology Society (EMBC).

[56] Gholamhosseini H., Meintjes A., Baig M.M., Lindén M. (2016). Smartphone-based Continuous Blood Pressure Measurement Using Pulse Transit Time. Studies in Health Technology and Informatics.

[57] Schoot T.S., Weenk M., van de Belt T.H., Engelen L.J., van Goor H., Bredie S.J. (2016). A new cuffless device for measuring blood pressure: A real-life validation study. J. Med. Internet Res..

[58] Jain M., Kumar N., Deb S. An affordable cuff-less blood pressure estimation solution. Proceedings of the 38th Annual International Conference of the IEEE Engineering in Medicine and Biology Society (EMBC).

[59] Kachuee M., Kiani M.M., Mohammadzade H., Shabany M. (2016). Cuffless blood pressure estimation algorithms for continuous health-care monitoring. IEEE Trans. Biomed. Eng..

[60] Tang Z., Tamura T., Sekine M., Huang M., Chen W., Yoshida M., Sakatani K., Kobayashi H., Kanaya S. (2017). A Chair–Based Unobtrusive Cuffless Blood Pressure Monitoring System Based on Pulse Arrival Time. IEEE J. Biomed. Health Inform..

[61] Seeberg T.M., Orr J.G., Opsahl H., Austad H., Røed M.H., Dalgard S.H., Houghton D., Jones D.E., Strisland F. (2017). A Novel Method for Continuous, Noninvasive, Cuff-Less Measurement of Blood Pressure: Evaluation in Patients With Nonalcoholic Fatty Liver Disease. IEEE Trans. Biomed. Eng..

[62] Janjua G., Guldenring D., Finlay D., McLaughlin J. Wireless chest wearable vital sign monitoring platform for hypertension. Proceedings of the 39th Annual International Conference of the IEEE Engineering in Medicine and Biology Society (EMBC).

[63] Liu S.-H., Cheng D.-C., Su C.-H. (2017). A Cuffless Blood Pressure Measurement Based on the Impedance Plethysmography Technique. Sensors.

[64] Chen Y., Cheng S., Wang T., Ma T. Novel blood pressure estimation method using single photoplethysmography feature. Proceedings of the 39th Annual International Conference of the IEEE Engineering in Medicine and Biology Society (EMBC).

[65] Ahmaniemi T., Rajala S., Lindholm H., Taipalus T. Pulse arrival time measurement with coffee provocation. Proceedings of the 39th Annual International Conference of the IEEE Engineering in Medicine and Biology Society (EMBC).

[66] Zhang Q., Zhou D., Zeng X. (2017). Highly wearable cuff-less blood pressure and heart rate monitoring with single-arm electrocardiogram and photoplethysmogram signals. Biomed. Eng. Online.

[67] Lin W., Wang H., Samuel O.W., Li G. Using a new PPG indicator to increase the accuracy of PTT-based continuous cuffless blood pressure estimation. Proceedings of the 39th Annual International Conference of the IEEE Engineering in Medicine and Biology Society (EMBC).

[68] Bhattacharya T., Gupta A., Singh S.T., Roy S., Prasad A. Robust motion artefact resistant circuit for calculation of Mean Arterial Pressure from pulse transit time. Proceedings of the 39th Annual International Conference of the IEEE Engineering in Medicine and Biology Society (EMBC).

[69] Ding X., Yan B.P., Zhang Y.-T., Liu J., Zhao N., Tsang H.K. (2017). Pulse transit time based continuous cuffless blood pressure estimation: A new extension and a comprehensive evaluation. Sci. Rep..

[70] Pflugradt M., Geissdoerfer K., Goernig M., Orglmeister R. (2017). A Fast Multimodal Ectopic Beat Detection Method Applied for Blood Pressure Estimation Based on Pulse Wave Velocity Measurements in Wearable Sensors. Sensors.

[71] Ding X.-R., Yan B.P., Yuan-Ting Z., Jing L., Peng S., Ni Z. Coherence analysis of invasive blood pressure and its noninvasive indicators for improvement of cuffless measurement accuracy. Proceedings of the Annual International Conference of the IEEE Engineering in Medicine and Biology Society.

[72] Xu J., Jiang J., Zhou H., Yan Z. A novel Blood Pressure estimation method combing Pulse Wave Transit Time model and neural network model. Proceedings of the Annual International Conference of the IEEE Engineering and Biology Society.

[73] Lo F.P., Li C.X., Jiankun W., Jiyu C., Meng M.Q. Continuous systolic and diastolic blood pressure estimation utilizing long short-term memory network. Proceedings of the Annual International Conference of the IEEE Engineering in Medicine and Biology Society.

[74] Rajala S., Lindholm H., Taipalus T. (2018). Comparison of photoplethysmogram measured from wrist and finger and the effect of measurement location on pulse arrival time. Physiol. Meas..

[75] Wang Y.-J., Chen C.-H., Sue C.-Y., Lu W.-H., Chiou Y.-H. (2018). Estimation of Blood Pressure in the Radial Artery Using Strain-Based Pulse Wave and Photoplethysmography Sensors. Micromachines.

[76] Lin W.-H., Wang H., Samuel O.W., Liu G., Huang Z., Li G. (2018). New photoplethysmogram indicators for improving cuffless and continuous blood pressure estimation accuracy. Physiol. Meas..

[77] Kim H., Park Y., Ko Y., Mun Y., Lee S., Ko H. (2018). Biosignal integrated circuit with simultaneous acquisition of ECG and PPG for wearable healthcare applications. Technol. Health Care.

[78] Sharifi I., Goudarzi S., Khodabakhshi M.B. (2018). A novel dynamical approach in continuous cuffless blood pressure estimation based on ECG and PPG signals. Artif. Intell. Med..

[79] Ahmaniemi T., Rajala S., Lindholm H., Taipalus T., Müller K. Variations of Heart Rate, Pulse Arrival Time and Blood Pressure in a Versatile Laboratory Protocol. Proceedings of the 40th Annual International Conference of the IEEE Engineering in Medicine and Biology Society (EMBC).

[80] Liang Y., Chen Z., Ward R., Elgendi M. (2018). Hypertension assessment via ECG and PPG signals: An evaluation using MIMIC database. Diagnostics.

[81] Xu Y., Ping P., Wang D., Zhang W. (2018). Analysis for the Influence of ABR Sensitivity on PTT-Based Cuff-Less Blood Pressure Estimation before and after Exercise. J. Healthc. Eng..

[82] Chen Y., Shi S., Liu Y.-K., Huang S.-L., Ma T. (2018). Cuffless blood-pressure estimation method using a heart-rate variability-derived parameter. Physiol. Meas..

[83] Feng J., Huang Z., Zhou C., Ye X. (2018). Study of continuous blood pressure estimation based on pulse transit time, heart rate and photoplethysmography-derived hemodynamic covariates. Australas. Phys. Eng. Sci. Med..

[84] Lillie J.S., Liberson A.S., Borkholder D.A. (2016). Improved Blood Pressure Prediction Using Systolic Flow Correction of Pulse Wave Velocity. Cardiovasc. Eng. Technol..

[85] Pickering T.G. (2003). What will replace the mercury sphygmomanometer?. Blood Press. Monit..

[86] Liang Y., Abbott D., Howard N., Lim K., Ward R., Elgendi M. (2019). How effective is pulse arrival time for evaluating blood pressure? Challenges and recommendations from a study using the MIMIC database. J. Clin. Med..

[87] Mukkamala R., Hahn J.-O., Inan O.T., Mestha L.K., Kim C.-S., Töreyin H., Kyal S. (2015). Toward ubiquitous blood pressure monitoring via pulse transit time: Theory and practice. IEEE Trans. Biomed. Eng..

[88] Mukkamala R., Hahn J.-O. (2017). Toward ubiquitous blood pressure monitoring via pulse transit time: Predictions on maximum calibration period and acceptable error limits. IEEE Trans. Biomed. Eng..

[89] Liang Y., Chen Z., Ward R., Elgendi M. (2018). Photoplethysmography and Deep Learning: Enhancing Hypertension Risk Stratification. Biosensors.

[90] Liang Y., Elgendi M., Chen Z., Ward R. (2018). An optimal filter for short photoplethysmogram signals. Sci. Data.

[91] Waugh W., Allen J., Wightman J., Sims A.J., Beale T.A. (2018). Novel signal noise reduction method through cluster analysis, applied to photoplethysmography. Comput. Math. Methods Med..

[92] Elgendi M. (2016). Optimal Signal Quality Index for Photoplethysmogram Signals. Bioengineering.

[93] IEEE Standard Association (2014). IEEE Standard for Wearable Cuffless Blood Pressure Measuring Devices. IEEE Std..

